# 

**Published:** 1851-10

**Authors:** 


### CONTENTS.

Article I.—Treatment of Dental Caries, complicated with Disorders of the Pulp and Peridental Membrane. By Robert Arthur, D. D. S., . . . . . . . . . 3

Article II.—An Essay on Artistic Dentistry. By A. Hill, D. D. S. 20

Article III.—Experiments on the Electro-Galvanic Process for Depositing Plates for Artificial Teeth. By A. Hill, D. D. S. 34

Article IV.—An Essay on Professional Empiricism. By A. C. Hawes, D. D. S. . . . . . . . 38

Article V.—Report on American Literature. By Robert Arthur, D. D. S. . . . . . . . . . . 43

Article VI.—Dental Education. By Charles W. Ballard, M. D., 59

Article VII.—Filling Teeth when the Lining Membraue is Exposed. By Chapin A. Harris, M. D. . . . . 72

Article VIII.—Historical Review of the Progress of Dental Surgery in the United States, . . . . . . 92

Article IX.—Cheap Artificial Teeth, and Cheap Dental Operations in England, . . . . . . . 101

Article X.—Cases of Cancrum Oris. By J. L. Levison, of Eng. 107

Article XI.—Adjusting Forcep. By G. F. J. Colburn, . 113

### SELECTED ARTICLES.

Article XII.—New Preparations of Gold for Stopping Carious Teeth. By John Tomes, F. R S., Surgeon Dentist to the Middlesex Hospital, . . . . . . 115

Article XIII.—An Instrument for Applying Electric Heat in Dental Operations. By George Waite, M. R. C. S., Surgeon Dentist. 126

Article XIV.—On the Destruction of the Dental Pulp by the Heat of Electricity. By Thomas H. Harding, Esq., . . 128

Article XV.—On Teething. By Mr. A. Canton, . . 131

Article XVI.—Remarks on a Peculiar Appearance observed in the Gums of Consumptive Patients. By T. Thompson, F. R. S. 138

Article XVII.—On the use of Impure Gold for Dental Purposes. By W. Imrie, Surgeon Dentist. . . . . . 140

Article XVIII. The Austrian Remedy for Cholera. . . 144

Article XIX.—Orbital Exostosis, . . . . 146

Article XX.—Instrument for Arresting Epistaxis. By M. Gabriel, 147

Article XXI.—On the Use of the Nitrate of Silver in Lacerated Wound of the Face, . . . . . 148

Article XXIII—A Professional and Technical Puzzle, . . 149

### BIBLIOGRAPHICAL.

Intermarriage. By Alexander Walker, . . . . . 150

Elements of General and Pathological Anatomy. By David Craige, M. D., F. R. S. E., &c. . . . . . . 153

The Microscopist. By Joseph H. Wythers, M. D. . . . 154

Annual Announcement of the Med. Faculty of McGill Col. Montreal, 154

Seventh Annual Announcement of the Ohio Col. of Dental Surgery, 155

Annual Announcement of the Rush Medical Col. of Chicago, Ill. 155

Annual Announcement of the Medical Department of Penn. College, 155

A new Sign Language for Deaf Mutes, . . . 156

A Plain Treatise on Dental Science and Practice. By S. M. Shepherd, 156

Report on the Medical Department of the University of Penn. . 156

Washington University of Baltimore, Medical Department, . 157

### EDITORIAL DEPARTMENT.

American Society of Dental Surgeons, . . . . . . . 157

Quacks Universally Advertise, . . . . . . . . . . 166

Osseous Union of Two Temporary Teeth, . . . . . . 167

Medical Appointment, . . . . . . . . . . . . 168

Ohio College of Dental Surgery, . . . . . . . . . 168

Omission, . . . . . . . . . . . . . 168

Dental Times, . . . . . . . . . . . . . 168

